# Small Bowel in Vagina: A Case for Pelvic Exams

**DOI:** 10.7759/cureus.17412

**Published:** 2021-08-24

**Authors:** Cody F Newell, Neil P Larson, Michael J Yoo

**Affiliations:** 1 Emergency Medicine, Brooke Army Medical Center, Fort Sam Houston, USA

**Keywords:** vaginal cuff dehiscence, pelvic exam, abdominal pain, dehiscence, hysterectomy

## Abstract

Vaginal cuff dehiscence with small bowel evisceration is a serious but uncommon postoperative complication of total laparoscopic hysterectomies. The severity of surgical site dehiscence can range from small, partial to full-thickness wound dehiscence, manifesting with acute abdominal pain, vaginal bleeding, or discharge, and is often precipitated by sexual intercourse. While imaging, including a pelvic ultrasound and computed tomography (CT), may help in undifferentiated acute abdominal pain, vaginal cuff dehiscence remains a clinical diagnosis found on physical exam. Because vaginal cuff dehiscence is a clinical diagnosis, sparse data exist regarding radiographic sensitivities and specificities in the identification of vaginal cuff dehiscence. Despite the increasing literature suggesting that pelvic exams are invasive with often limited utility, the authors argue that pelvic exams remain essential in identifying complications of hysterectomies. The authors present a case of a 40-year-old woman with acute abdominal pain found to have loops of small bowel in the vaginal vault, discovered only on physical exam after negative CT and ultrasound imaging.

## Introduction

Dehiscence of the vaginal cuff is a rare but serious complication after laparoscopic total hysterectomies, with ranges between an incidence of 0.14%-4.1% varying by multiple factors including surgeon experience, closure technique, etc.; however, the largest studies to date cite rates less than 0.5% [1]. Although it may present with abdominal pain, vaginal bleeding, and discharge, at the most extreme, complete dehiscence with evisceration of the small bowel and other intraperitoneal organs has an incidence up to 1.2% with an associated mortality rate of 5.6% [1,2]. While lab work and imaging can assist with undifferentiated acute abdominal pain, obtaining an accurate surgical history and physical exam, including a pelvic and bimanual exam, is essential in identifying this post-surgical complication. We present a case of a 40-year-old woman with acute abdominal pain after engaging in sexual intercourse, found to have loops of small bowel in her vaginal vault, identified solely on physical exam.

## Case presentation

A 40-year-old woman with a history of a total laparoscopic hysterectomy four months prior for bleeding fibroids presented to the emergency department (ED) with acute onset, generalized abdominal pain for one hour that began after having consensual receptive vaginal intercourse. The patient stated that her postoperative course was uncomplicated, denied recent illnesses, recent changes in her diet, or new medications. Her review of systems, however, was positive for mild nausea and 10 out of 10 abdominal pain. The patient denied any vaginal bleeding or discharge.

On arrival, the patient’s vital signs included: blood pressure of 120/82 mmHg, heart rate of 78 beats per minute, the temperature of 98.9 degrees Fahrenheit, 16 respirations per minute, and saturating 100% on room air. The patient’s exam was notable for severe, generalized abdominal tenderness, mild guarding without rebound tenderness, and no costovertebral angle tenderness. A pelvic exam was declined by the patient until alternative diagnostic studies were obtained. The patient was administered 4 mg of intravenous (IV) morphine and 4 mg of IV ondansetron with moderate relief of symptoms.

**Figure 1:**
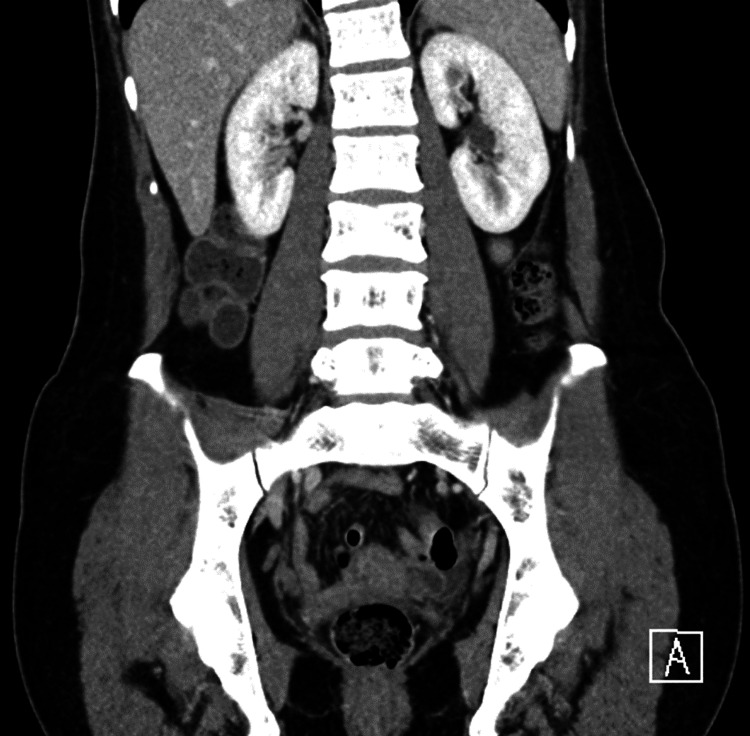
Coronal section of a contrasted computed tomography of the abdomen and pelvis, without acute findings.

**Figure 2:**
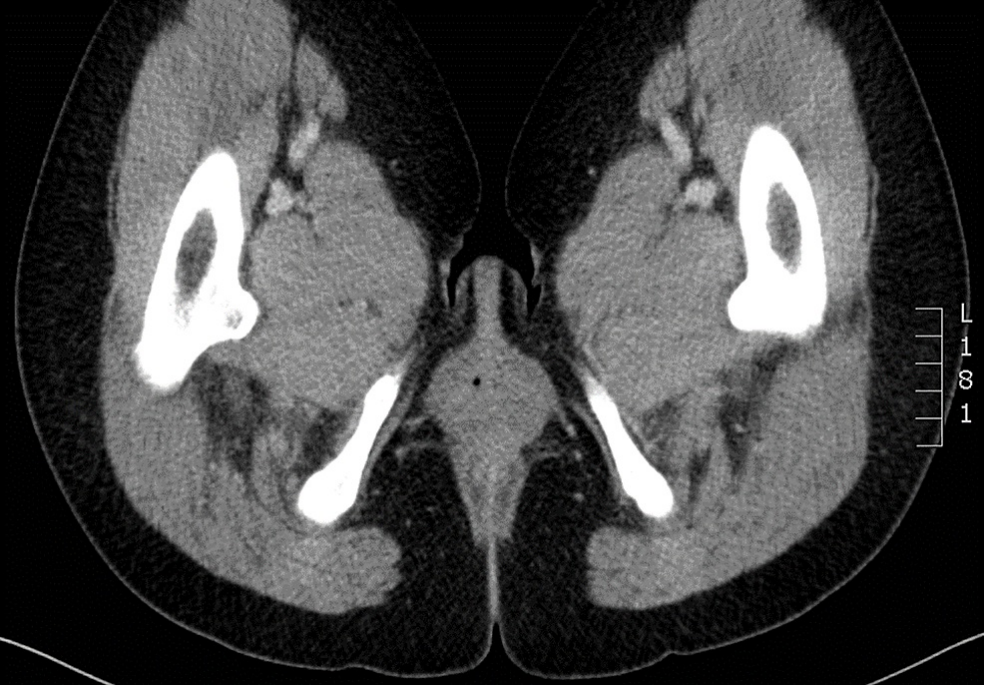
Axial section of a contrasted computed tomography of the abdomen and pelvis, with no acute findings.

A complete blood cell count, complete metabolic panel, and urinalysis were all unremarkable. A transvaginal ultrasound re-demonstrated her post-hysterectomy status but otherwise revealed no acute findings. Please note that the local hospital practice is to allow the patient to self insert the endocavitary probe to minimize discomfort prior to the ultrasound technician performing the exam. A computed tomography (CT) of the abdomen and pelvis was also obtained but revealed no acute findings (Figures 1, 2).

The patient returned from CT imaging with recurrence of pain and nausea, requiring re-administration of IV morphine and ondansetron. After notifying the patient of a non-diagnostic workup thus far, the patient agreed to a pelvic exam, which was notable for loops of small bowel with active peristalsis in the vaginal vault. Small amounts of microtrauma were noted on the side of an intact small bowel. A subsequent bimanual exam revealed a large defect in the anterior vaginal wall communicating with her peritoneum with leakage of approximately 5 cc of serosanguinous fluid. After consulting obstetrics and gynecology, the patient went to the operating room for an emergent laparoscopy. Chart review of the operative report revealed complete vaginal cuff dehiscence with small bowel evisceration. The patient was discharged after an unremarkable postoperative course.

## Discussion

Over half a million hysterectomies are performed annually in the United States, making it one of the most common surgical procedures, with indications ranging from uterine fibroids, dysfunctional vaginal bleeding, endometriosis, uterine prolapse, infection, and pain [3,4]. Although the rates of the laparoscopic approach are increasing in an attempt to decrease recovery times and rates of infection, most hysterectomies are approached trans-abdominally (64%), over vaginally (22%), and laparoscopically (14%) [3]. In cases of total hysterectomies, the cervix is additionally separated from the apex of the vagina with subsequent cuff closure, using a running suture [5].

The severity of dehiscence ranges from partial thickness (reflecting the depth of dehiscence) and incomplete dehiscence (reflecting the length of dehiscence) to full thickness and complete dehiscence along the vaginal surgical site [6]. At the most extreme of the complication spectrum, patients can have associated evisceration of intra-abdominal organs, typically the small bowel, through the sites of dehiscence [5]. The incidence of post-hysterectomy vaginal cuff dehiscence in the largest studies finds the laparoscopic approach as the highest, ranging from 0.11% to 0.75%, compared to an abdominal approach of 0.05-0.38%, and vaginal approach of 0.02-0.11% [1]. Rates of evisceration associated with vaginal cuff dehiscence occur in up to 1.2% of all hysterectomy cases [1].

Aside from a laparoscopic approach, additional risk factors for vaginal cuff dehiscence include older age, prior gynecologic surgeries, and atrophy of the vagina prior to the procedure [6]. As with any surgical procedure, conditions that affect wound healing are also risk factors for dehiscence - these include steroid use, cancers especially with active radiation therapy, poor nutrition status, postoperative infection, and increase in intra-abdominal pressure such as post-operative constipation [6,7]. In younger women, sexual intercourse has been cited as a common inciting factor, in up to 76% of cases [6].

Minimal data exist regarding the most frequent postoperative timeframe for presentation, but patients typically present within 24 hours of symptom onset [7]. Most commonly, patients with dehiscence experience abdominal pain, vaginal bleeding or discharge, and a sensation of vaginal pressure [7]. Vaginal cuff dehiscence with small bowel evisceration is a clinical diagnosis based on a physical exam, including a speculum and bimanual exam [6]. Once identified, the patient should be kept in a supine or Trendelenburg position to prevent worsening of the evisceration, while emergent obstetric and gynecologic consultation is made for operative repair [6].

Several studies have evaluated the utility of pelvic exams in the emergency department in an attempt to improve patient comfort [8-10]. Although data suggest that foregoing the pelvic exam does not significantly worsen patient outcomes and can be unreliable, we argue that despite the availability of advanced imaging and lab work, a pelvic examination is irreplaceable in any patient with concern for a postoperative complication. As evidenced in our case, reliance on advanced imaging and failure to utilize a pelvic exam to re-evaluate the surgical site can result in failure to identify dehiscence and evisceration.

## Conclusions

Vaginal cuff dehiscence with small bowel evisceration is a rare postoperative complication of total hysterectomies, with a laparoscopic approach increasing the patient’s risk. Postoperative patients who present with acute onset lower abdominal pain, vaginal bleeding, or discharge, especially after intercourse, should be evaluated for surgical site dehiscence with a speculum and bimanual exam. Any suspicion for vaginal cuff dehiscence with or without evisceration requires emergent consultation with obstetrics and gynecology for operative repair.
